# Risk factors for the development of acute lung injury in patients with infectious pneumonia

**DOI:** 10.1186/cc11247

**Published:** 2012-03-14

**Authors:** Marija Kojicic, Guangxi Li, Andrew C Hanson, Kun-Moo Lee, Lokendra Thakur, Jayanth Vedre, Adil Ahmed, Larry M Baddour, Jay H Ryu, Ognjen Gajic

**Affiliations:** 1 The Division of Pulmonary and Critical Care Medicine, Department of Medicine, Mayo Clinic College of Medicine, 200 First Street SW, Rochester, MN 55905, USA; 2 Urgent Pulmonology Department, The Institute for Pulmonary Diseases of Vojvodina, Institutski put 4, Sremska Kamenica 21204, Serbia; 3 Department of Pulmonary Medicine, Guang An Men Hospital, China Academy of Chinese Medical Science, 5 BeiXianGe Street, Beijing 100053, China; 4 The Division of Biostatistics, Department of Health Sciences Research, Mayo Clinic College of Medicine, 200 First Street SW, Rochester, MN 55905, USA; 5 Department of Anesthesiology, Paik Hospital, College of Medicine, InJe University, Gaegeum 2-dong, Busanjin-gu, Busan 614-735, South Korea; 6 The Division of Infectious Diseases, Department of Medicine, Mayo Clinic College of Medicine, 200 First Street SW, Rochester, MN 55905, USA

## Abstract

**Introduction:**

Although pneumonia has been identified as the single most common risk factor for acute lung injury (ALI), we have a limited knowledge as to why ALI develops in some patients with pneumonia and not in others. The objective of this study was to determine frequency, risk factors, and outcome of ALI in patients with infectious pneumonia.

**Methods:**

A retrospective cohort study of adult patients with microbiologically positive pneumonia, hospitalized at two Mayo Clinic Rochester hospitals between January 1, 2005, and December 31, 2007. In a subsequent nested case-control analysis, we evaluated the differences in prehospital and intrahospital exposures between patients with and without ALI/acute respiratory distress syndrome (ARDS) matched by specific pathogen, isolation site, gender, and closest age in a 1:1 manner.

**Results:**

The **s**tudy included 596 patients; 365 (61.2%) were men. The median age was 65 (IQR, 53 to 75) years. In total, 171 patients (28.7%) were diagnosed with ALI. The occurrence of ALI was less frequent in bacterial (*n *= 99 of 412, 24%) compared with viral (*n *= 19 of 55, 35%), fungal (*n *= 39 of 95, 41%), and mixed isolates pneumonias (*n *= 14 of 34, 41%; *P *= 0.002). After adjusting for baseline severity of illness and comorbidities, patients in whom ALI developed had a markedly increased risk of hospital death (OR_adj _9.7; 95% CI, 6.0 to 15.9). In a nested case-control study, presence of shock (OR, 8.9; 95% CI, 2.8 to 45.9), inappropriate initial antimicrobial treatment (OR, 3.2; 95% CI, 1.3 to 8.5), and transfusions (OR, 4.8; 95% CI, 1.5 to 19.6) independently predicted ALI development.

**Conclusions:**

The development of ALI among patients hospitalized with infectious pneumonia varied among pulmonary pathogens and was associated with increased mortality. Inappropriate initial antimicrobial treatment and transfusion predict the development of ALI independent of pathogen.

## Introduction

Despite recent improvements in supportive treatment, acute lung injury (ALI) remains a devastating syndrome, with pneumonia as the most common predisposing condition [1]. Although recent data demonstrated temporal improvement in survival, the mortality in ALI patients still remains high [2,3].

Because therapeutic options are limited and the majority of intervention strategies are focused on supportive treatment, emphasis has been placed on identifying patients who are at higher risk for ALI [4,5]. Patients with ALI represent a heterogeneous group of patients with regard to predisposing conditions [6] that differ in pathophysiologic changes [7,8], clinical and radiologic [9] characteristics, as well as treatment options [10,11], but the data on specific risk factors in subsets of patients according to predisposing causes are limited. Although sepsis, particularly pulmonary in origin, is the most common underlying risk factor for ALI, in only a small proportion of hospitalized patients with pneumonia does the complication develop (< 10%) [4]. Defining risk factors associated with the development of ALI in patients with infectious pneumonia is challenging because virulence factors of different pathogens have been implicated in causing lung damage [12,13]. In patients with pneumonia, the relation between ALI and specific pathogens has been described mainly in case series and case reports of acute respiratory distress syndrome (ARDS) in patients with *Legionella *and certain types of viral and fungal pneumonia, suggesting that some pathogens are particularly prone to induce lung injury. A recent study of critically ill patients demonstrated that pulmonary infection is associated with a higher risk of developing ARDS, as compared with infections at nonpulmonary sites [14]. Systematic data regarding occurrence, risk factors, and outcome of ALI/ARDS in patients with infectious pneumonia are lacking.

The objective of the present study was to determine the frequency and outcome of ALI in a retrospective cohort of hospitalized patients with microbiology-proven pneumonia and to identify prehospital and hospital exposures that may predict the development of ALI, independent of pathogen.

## Materials and methods

We used validated queries of the Mayo Clinic electronic medical record database (Mayo Clinic Life Sciences System) [15,16] to identify consecutive patients with microbiologically positive pneumonia who were hospitalized at the two Mayo Clinic Rochester hospitals between January 1, 2005, and December 31, 2007. The Institutional Review Board approved the study protocol and waived the need for informed consent in this observational study. To be included in the study, patients had to be at least 18 years of age and have a diagnosis of pneumonia with identified pathogens according to the *International Classification of Diseases, 9th Revision, Clinical Modification (ICD-9-CM) *(ICD codes 480.0-480.8, 481-482.89, 483-484.8, 487.0, 114.0, 115.5, 116.0 and 136.3). If a patient had more than one episode that met inclusion criteria, only the first episode was included. Electronic medical records, including portable digital chest radiographs and microbiology reports, were independently reviewed to confirm the diagnosis of pneumonia and pathogen isolation. Pneumonia was defined as a new or progressive infiltrate as seen on a chest radiograph or CT scan along with a high clinical suspicion of pneumonia, defined with at least one of the following: fever (> 38°C or > 100.4°F), leukopenia (< 4,000 WBC/mm^3^) or leukocytosis (> 12,000 WBC/mm^3^), altered mental status with no other recognized cause (for adults older than 70 years), and at least two of the following: (a) new onset of purulent sputum, or change in character of sputum, or increased respiratory secretions, or increased suctioning requirements, (b) new onset or worsening cough, or dyspnea, or tachypnea, (c) rales or bronchial breath sounds, and (d) worsening gas exchange, increased oxygen requirements, or increased ventilation demand [17,18]. The onset of pneumonia (the time of diagnosis of pneumonia) was defined by the first recorded time of any criterion when these criteria were met.

### Microbiological etiology

Patients were included if one or more respiratory pathogens were recovered from: (a) an uncontaminated specimen (blood, pleural fluid, transtracheal aspirate, transthoracic aspirate, or surgical lung biopsy specimen); (b) positive serology defined as elevated immunoglobulin M (IgM) antibodies or fourfold increases in IgG antibody titers; (c) positive urinary antigen test; (d) positive polymerase chain reaction (PCR); (e) semiquantitative cultures of a lower respiratory tract sample (endotracheal aspirate, bronchoalveolar lavage (BAL), or protected specimen brush); (f) or expectorated sputum culture.

We identified ALI patients with a validated electronic syndrome surveillance tool (ALI "sniffer") that was developed for early recognition of patients meeting inclusion criteria for ARDS-net trials. The negative predictive value 99.6% (95% CI, 99.3 to 99.8) of the electronic alert had been determined in a previous study against the gold standard of prospective assessment by trained intensivist researchers, blinded to the ALI electronic alert. The details of the ALI electronic alert have been previously published [16]. All patients with a positive ALI "sniffer" were reassessed and ALI diagnosed by an independent review of portable chest radiographs, arterial blood gases, and hemodynamic parameters based on American/European consensus conference definition [19]. For adequate interpretation of radiologic studies in the diagnosis of ALI, the abstractors reviewed a structured ALI tutorial before study onset. Interrater reliability for diagnosing ALI was assessed in previous studies (kappa value of 0.8) [20]. The timing of ARDS was determined by the first recorded time of either criterion when both criteria (PaO_2_/FIO_2_, bilateral infiltrates) were met.

To identify the risk factors for ALI development, in a subsequent nested case-control study, patients who developed ALI more than 6 hours after pneumonia onset were matched to patients at risk who did not develop ALI, based on specific pathogen, isolation site, gender, and closest age in a 1:1 matching. If an appropriate control was not found for a specific pathogen, matching was for the same genus, family, and finally pathogen group (G-, G+, atypical bacteria, viruses, and fungi). Patients with coinfections were matched to appropriate controls in the same fashion (all pathogens were matched). Because preliminary data suggested increased risk of ALI among *Pneumocystis jirovecii *pneumonia, these cases were matched to specific pathogen, regardless of coinfection. The matching was conservative to provide as perfect as possible matches. Among 171 cases of ALI, 118 were diagnosed with ALI more than 6 hours after pneumonia onset, of whom 112 were matched according these criteria. We extracted data from the preexisting hospital electronic database, which included integrated microbiologic and susceptibility results, vital signs as well as laboratory parameters, comorbidities, and use of medications. Risk factors were compared between patients who developed ALI and matched controls. For quality assurance, we performed random checks of electronic database entries. Standard definitions were used for shock [21], inappropriate antimicrobial treatment [22], and transfusions [23]. We used the Pneumonia severity index (PSI) [24] to assess baseline severity of illness and the Charlson score [25] as a measure of comorbidities. Arterial oxygen saturation measured by pulse oximetry (SpO_2_) was recorded if there was a delay in obtaining arterial blood gas analysis, and the SpO_2_/FIO_2 _ratio was used to substitute PaO_2_/FIO_2 _ratio (SpO_2_/FIO_2 _< 315 corresponds to PaO_2_/FIO_2 _< 300) [26]. To assure similar exposure time to possible risk factors between cases and controls, the exposure time for each control was matched to the exposure time of the corresponding case. That means that data on possible intrahospital exposures of cases were collected from onset of pneumonia to onset of ALI, whereas in controls, data were tabulated for the same number of hours subsequent to the onset of pneumonia.

We excluded patients with pneumonectomy and ventilator-associated pneumonia (VAP). VAP patients were excluded to eliminate the possibility of including patients with pneumonia complicating ALI and to limit effect-cause bias. Patients with potentially contaminated blood culture (bacteremia cases with a single positive blood culture of coagulase-negative staphylococci) were also excluded from the analysis.

### Statistical analysis

Continuous variables were compared by using the Student *t *test for normally distributed variables and the Wilcoxon rank-sum test for nonnormally distributed variables. The χ^2 ^or Fisher exact test was used to compare categoric variables. In a retrospective cohort of pneumonia patients, the association between pathogens and ALI occurrence, and ALI and hospital mortality was assessed in univariate followed by multivariate logistic regression analysis, after adjusting for baseline severity of illness. In a subsequent nested case-control study of 112 ALI cases and 112 matched controls, paired parametric and nonparametric testing were used as appropriate, followed by a conditional logistic regression to investigate the relation between ALI and specific baseline characteristics and in-hospital exposures. Selection of the variables for a conditional logistic regression model was done, considering both clinical plausibility, and statistical criteria (significance, colinearity, and interaction). All *P *values of < 0.05 were considered to indicate statistical significance. SAS statistical software (SAS version 9; SAS Institute, Inc., Cary, NC, USA) was used for statistical analysis.

## Results

**Figure 1:**
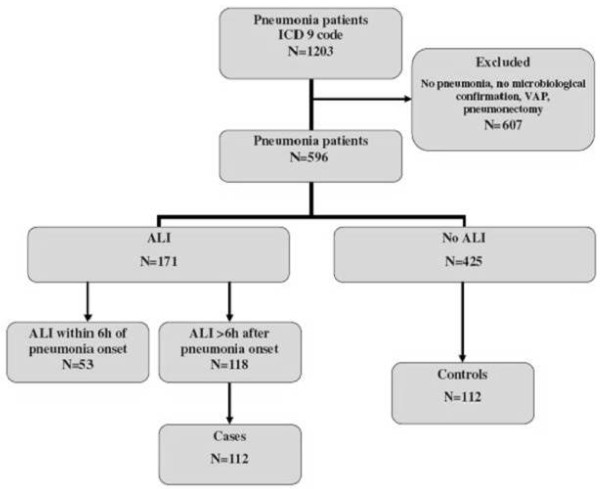
**Study flow diagram**.

Figure 1 shows the flow of study participants. The cohort included 596 patients; 365 (61.2%) were men. The median age was 65 (interquartile range (IQR), 53 to 75) years. The etiology of pneumonia was mostly bacterial (*n *= 412; 69%), followed by fungal (*n *= 95; 16%), viral (*n *= 55; 9%), and mixed pathogens (*n *= 34; 6%). Co-infections were present in 131 (22%) cases. The pathogens were most commonly isolated from sputum in 206 (28.2%), in tracheal aspirate 151 (20.6%), in bronchoalveolar lavage (BAL) fluid 126 (17.3%), in blood 92 (12.6%), and in bronchial washings 46 (6.3%).

Baseline characteristics of patients are presented in Table 1. One hundred and seventy-one patients (28.7%) were diagnosed with ALI. ALI less commonly occurred in patients with community-acquired pneumonia (CAP) (*n *= 61; 21.8%) compared with those with healthcare-associated (HCAP) (*n *= 53; 31.6%; *P *= 0.02) and hospital-acquired pneumonias (HAP) (*n *= 57, 38.5%; *P *< 0.001). Among 448 patients who had evidence of pneumonia at the time of hospital admission, 43 (9.6%) presented with ALI on admission, of whom 21 (49%) were transferred from another hospital; in the remainder, the median time to development of ALI was 2 (IQR, 1 to 3) days.

**Table 1:** Baseline characteristics of the cohort

	*N *= 596
Age, median (IQR)	64.5 (53-75)
Male gender, *n *(%)	365 (61)
Pneumonia severity index score, median (IQR)	119 (94-146)
Charlson comorbidity score, median (IQR)	2 (1-4)
Pneumonia type, *n *(%)	
CAP	280 (47)
HCAP	168 (28)
HAP	148 (25)

CAP, community-acquired pneumonia; HAP, hospital-acquired pneumonia; HCAP, healthcare-associated pneumonia; IQR, interquartile range.

**Figure 2:**
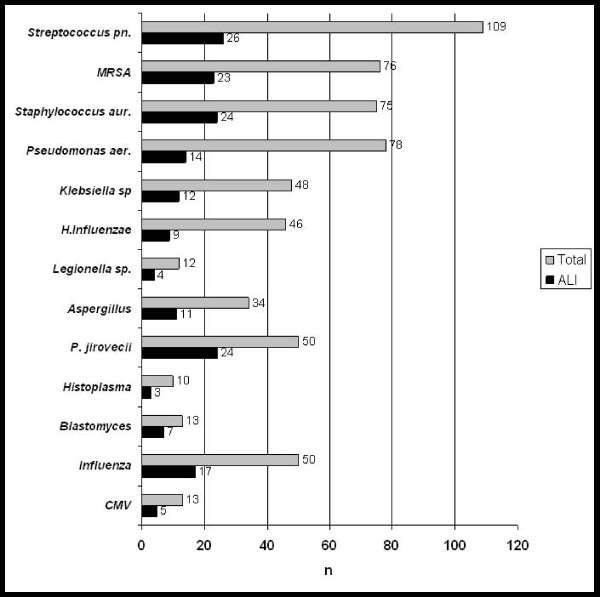
**Frequency of ALI among the most commonly isolated pathogens**. *Coinfections included.

The occurrence of ALI was less frequent in bacterial pneumonias (*n *= 99 of 412; 24%) compared with viral (*n *= 19 of 55; 35%), fungal (*n *= 39 of 95; 41%), and mixed isolates pneumonias (*n *= 14 of 34; 41%) (*P *= 0.002). Figure 2 displays the frequency of ALI according to the most common pathogens. Similar results were obtained when coinfections were excluded from the analysis. When the analysis was restricted to bacteremic patients, Gram-positive and Gram-negative bacterial infections had a similar frequency of ALI (20% versus 15%; p = 0.75). All patients diagnosed with respiratory syncytial virus (RSV) pneumonia (four of four) developed ALI. Pneumonia due to *Pneumocystis jiroveci *and *Blastomyces *species was associated with increased risks of ALI when compared to *Streptococcus pneumoniae*, the most common cause of pneumonia, in both univariate (OR, 3.35; 95% CI, 1.5 to 7.61; OR, 4.41; 95% CI, 1.1 to 19.4, respectively) and multivariate analyses (OR, 3.8; 95% CI, 1.65 to 8.93; OR, 5.6; 95% CI, 1.3 to 26.2; respectively), after adjusting for baseline severity of illness (pneumonia severity index). Patients who developed ALI had a markedly increased risk of hospital death in both univariate (OR_adj _9.2; 95% CI, 5.8 to 14.8) and multivariate analysis after adjusting for baseline severity of illness and comorbidities (Charlson score) (OR_adj _9.7; 95% CI, 6.0 to 15.9).

The association between previously proposed risk factors for the development of ALI was examined in 112 patients who had no ALI at the time pneumonia was diagnosed and who were matched by specific pathogen, age, and gender. The characteristics of ALI cases and controls are presented in Table 2. Patient groups were similar in terms of comorbidities expressed as cumulative Charlson score. No difference was found in frequency of diabetes between cases and controls. Patients who developed ALI were more likely to present with bilateral infiltrates. Fifty-one patients (45.5%) that presented with unilateral infiltrate later progressed to ALI. ALI cases had lower SaO_2_/FiO_2 _at the baseline and higher baseline pneumonia severity index. Most of the patients with RSV and PCP infections were immunosuppressed (30 of 32). The median time to any antibiotic administration was similar in cases and controls, but inappropriate initial antimicrobial treatment was associated with increased risk of ALI (Table 2). The transfusion of fresh frozen plasma, platelets, and red blood cells was associated with ALI development.

**Table 2:** Univariate analysis of baseline characteristics and interventions in ALI cases and matched controls

	ALI cases *n *= 112	Controls *n *= 112	Odds ratio	*P *value
**Baseline characteristics**				
Age (years) Median (IQR)	64.5 (53.5-72.0)	62.0 (53.0-71.5)	1.00 (0.98-1.03)	0.65
Male gender *n *(%)	56 (50)	61 (55)	1.43 (0.68-3.3)	0.52
BMI Median (IQR)	26.7 (24.07-31.05)	26.4 (21.9-30.4)	1.00 (0.99-1.01)	0.48
Ever smoker *n *(%)	66 (59)	52 (46)	1.24 (0.96-1.65)	0.07
Alcohol abuse *n *(%)	13 (12)	9 (8)	1.28 (0.74-1.93)	0.37
Pneumonia type				0.007^a^
*n *(%)				
CAP	33 (29.5)	55 (49)	0.32 (0.15-0.63)	
HCAP	33 (29.5)	29 (26)	1.22 (0.66-2.27)	
HAP	46 (41)	28 (25)	2.10 (1.19-3.86)	
Charlson score Median (IQR)	3 (2-4)	2 (1-4)	1.0 (0.88-1.14)	0.26
Aspiration *n *(%)	8 (7.1)	4 (3.6)	1.84 (0.63-6.55)	0.25
Diabetes *n *(%)	25 (22)	33 (29)	0.71 (0.39-1.26)	0.24
Immunosuppression *n *(%)	52 (46.4)	50 (44.6)	1.13 (0.54-2.36)	0.73
Pneumonia severity index score Median (IQR)	115 (92-142)	101 (76-119)	1.02 (1.01-1.03)	< 0.001 ^a^
Shock *n *(%)	47 (42)	9 (8)	16.3 (5.47-79.04)	< 0.001 ^a^
Respiratory rate Median (IQR)	23 (20-28)	20 (18-24)	1.04 (1.00-1.08)	0.05
SaO_2_/FiO_2_ Median (IQR)	236 (107-336)	336 (247-448)	0.995 (0.993-0.997	< 0.001 ^a^
Bilateral infiltrates *n *(%)	61 (54.5)	31 (38.2)	3.67 (1.92-7.61)	< 0.001 ^a^
Pleural effusion *n *(%)	23 (20.5)	17 (15.4)	1.34 (0.68-2.69)	0.38
**Interventions**				
Time to antibiotics (hours) Median (IQR)	0 (0-9)	0 (0-3)	1.00 (0.99-1.02)	0.11
Appropriate initial antimicrobial treatment *n *(%)	62 (55.4)	85 (75.9)	0.29 (0.13-0.58)	< 0.001 ^a^
Any transfusion *n *(%)	38 (33.9)	15 (13.4)	4.53 (2.08-11.59)	< 0.001 ^a^
Platelets	15 (13.4)	4 (3.6)	6.50 (1.47-59.33)	0.0098 ^a^
Red blood cells	31 (27.7)	13 (11.6)	4.0 (1.59-11.96)	0.002 ^a^
Fresh frozen plasma	10 (8.9)	1 (0.9)	-	0.008 ^a^
Corticosteroids systemic *n *(%)	48 (42.9)	51 (45.5)	0.89 (0.51-1.55)	0.67
Mechanical ventilation *n *(%)	95 (85)	24 (21)	29.4 (10.2-141.17)	< 0.001 ^a^
Invasive	80 (71)	19 (17)	18.36 (7.24-66.69)	
Noninvasive	15 (14)	5 (4)	2.83 (1.13-8.25)	

BMI, Body mass index; CAP, community-acquired pneumonia; HAP, hospital-acquired pneumonia; HCAP, healthcare-associated pneumonia; IQR, interquartile range.^a^

a- statistically significant

When adjusted for baseline imbalances (shock, SpO_2_/FiO_2_, type of pneumonia) in a conditional logistic regression analysis, inappropriate antimicrobial treatment (OR, 3.2; 95% CI, 1.3 to 8.5) and transfusions (OR, 4.8; 95% CI, 1.5 to 19.6) were independently associated with the development of ALI (Table 3). The results were similar after adjustment for PSI (as a composite measure of baseline severity of illness) (Table 4).

**Table 3:** Conditional regression analysis of ALI risk factors

	OR	95% CI
Shock	8.9	2.8-45.9
171717	0.996	0.993-0.999
Inappropriate initial antimicrobial treatment	3.2	1.3-8.5
Any transfusion	4.8	1.5-19.6
HAP	1.9	0.8-4.5

HAP, hospital-acquired pneumonia.

**Table 4:** Conditional regression analysis of ALI risk factors

	OR	95% CI
PSI	1.01	1.00-1.03
Inappropriate initial antimicrobial treatment	3.1	1.5-7.0
Any transfusion	3.2	1.3-8.8
HAP	1.8	0.9-3.8

HAP, hospital-acquired pneumonia; PSI, pneumonia severity index.

Patients who developed ALI were more likely to be mechanically ventilated, had longer hospital length of stay, and had a markedly increased risk of hospital death (Additional file 1). In a *post hoc *analysis we explored the association between the prehospital use of certain medications (statins, ACE inhibitors, and antiplatelet drugs) that were found to modify development of ALI in previous studies. When adjusted for adequate antibiotics, transfusion, and PSI in conditional logistic regression analysis, the use of a statin was associated with a decreased risk of ALI (OR, 0.36; 95% CI, 0.14 to 0.92).

## Discussion

The results of this study suggest that ALI is common in hospitalized pneumonia patients with positive microbiologic diagnosis. In addition, patients with bacterial pneumonia had lower rates of ALI compared with those with fungal, viral, and mixed infections. When controlled for age, gender, and specific pathogen, independent predictors of ALI in pneumonia patients are baseline severity of illness, inappropriate initial antimicrobial treatment, and transfusion.

ALI rates in our study were higher compared with a previous study of hospitalized patients with clinically defined pneumonia by Ferguson and colleagues (10%) [4]. This could be due to the inclusion, in our study, of only patients with a high clinical suspicion of pneumonia and identified isolates, which has been described in fewer than 50% of hospitalized patients with pneumonia [27]. In addition, most of our patients had an infectious agent isolated from lower respiratory tract secretions, indicating that this population probably represents a group of more severely ill patients. A recent report of infection-related ARDS in patients with sepsis, pneumonia, and bacteremia yielded results similar to ours [14].

In our study, the main causes of pneumonia were *S. aureus *(with high percentage of MRSA), *S. pneumoniae and Pseudomonas aeruginosa*. Similar results were obtained by Kollef *et al. *[28]. Among the most common pathogens, no difference in the rates of ALI was found. We observed a higher proportion of ALI occurrence in patients with less common infections due to fungal and viral respiratory pathogens. The higher rate of ALI among these pathogen groups likely relates to *P. jiroveci*, and specific endemic fungi, including *Blastomyces *species and viral infections (RSV, cytomegalovirus). Possible explanations for this higher risk include delayed antimicrobial treatment, as these pathogens are often not treated with initial empiric antimicrobial coverage, as well as baseline characteristics of the patients, because these infections more commonly occur in those who are immunocompromised. Progression to ALI is likely a result of a complex interaction of patient immune status and specific pathogen [29]. Conversely, when adjusted for a specific pathogen, immunosupression did not influence progression to ALI. However, these results must be interpreted with caution because matching by specific pathogen is similar to matching by immune status or other baseline risk factors for these pathogens, which precludes analyzing the significance of these risk factors for developing ALI. Unfortunately, our study design does not allow further assessment of independent effects of a pathogen on risk for ALI development. Although numerous reports exist of *Legionella *pneumonia-induced ARDS, our study failed to confirm an increased risk of ALI in these patients. It is possible that early initiation of empiric antimicrobial coverage for atypical bacteria, as proposed in current pneumonia guidelines, has reduced this devastating complication in patients with *Legionella *pneumonia. We observed no case of ALI in the course of pneumonia caused by other "atypical" bacteria.

Several studies have shown that ALI is rarely present at the time of hospitalization and usually develops in hours to days after hospital admission [4,30]. The evolution of ALI could be influenced by both baseline characteristics of the patients (first hit) as well as a variety of intrahospital exposures (second hit) [31]. Certain prehospital exposures (alcohol, smoking) [32,33], medical errors (delayed shock resuscitation, delayed antibiotic treatment) [30], and iatrogenic exposures (plasma transfusion from alloimmunized donors, gastric aspiration, certain chemotherapeutic drugs) [23,34,35] have all been associated with development of ALI in hospitalized patients. Similar to the results of other studies [35], we observed higher baseline severity of illness among ALI cases. Considering baseline characteristics, patients with ALI were more often smokers, although statistical difference was not reached. This could be because we used patients' histories to obtain information on smoking. In a recent study by Hsieh [36], serum and urine nicotine metabolites identified considerably more active smokers than did smoking history. Active and passive cigarette smoking was found to be associated with ALI after severe blunt trauma, when a biomarker of tobacco smoking was used to assess smoking history [37]. Unlike several other studies [32,38], the history of alcohol abuse did not influence the risk of ALI. In addition, although the majority of studies implicated diabetes to have a protective role [39,40], no difference in number of diabetics was found between the cases and controls in our study.

The highest percentage of ALI was found among patients with healthcare-associated pneumonia (HCAP) and hospital-acquired pneumonia (HAP), suggesting that health-care-related exposures could have contributed to the development of ALI. When matched for age, gender, and pathogen characteristics, and adjusted for baseline severity of illness, the major interventions that were associated with an increased risk of ALI were transfusions and inappropriate initial antimicrobial therapy. Previous studies demonstrated an increased risk of ALI in patients with delayed treatment of infection [30]. The time of antibiotic initiation did not influence ALI development in our study, as most patients received antibiotics in the emergency department or immediately after admission; however, ALI was associated with inappropriate initial antimicrobial therapy. In patients with pneumonia, inappropriate initial antibiotic treatment noticeably increases the risk of hospital death [41,42]. The failure to provide adequate antibiotic treatments is more likely in HCAP and HAP, as Gram-negative bacteria and *S. aureus *are major pathogens in these patients [28]. In addition to antimicrobial resistance [41], it seems that patients with HCAP are often not empirically treated for these organisms, resulting in suboptimal antibiotic therapy and increased mortality [43]. Although shock was clearly associated with ALI development, the association of delayed goal-directed resuscitation could not be examined because of a relatively low number of shock patients among controls. The observed association between transfusion and lung injury has been demonstrated in number of studies [40,44,45]. The observed association between the use of statins and the decreased risk of ALI is consistent with the recent report by O'Neal *et al. *[46]. Contrary to a previous report in a population-based cohort of nonselected patients at risk of ALI [47], the use of antiplatelet agents in patients with infectious pneumonia was not associated with decreased risk of ALI.

Severe pneumonia is associated with high rates of morbidity and mortality [48-51]. The majority of these studies failed to account for ALI as well as its role in the course of pneumonia and patient outcomes. Our work demonstrated that the development of ALI is independently associated with increased mortality after adjusting for baseline severity of illness and comorbidities. This is particularly important because multiple factors influence ALI development and are potential targets for preventing this devastating syndrome.

Our study has several limitations. First, enrollment was limited to microbiologically proven pneumonia. Because the majority of patients with pneumonia do not undergo any microbial testing, some patients with pneumonia were excluded and may have biased our study population to more severe cases that underwent such evaluation. Diagnosis of atypical pneumonia is difficult because of the fastidious nature of atypical organisms; the sensitivity of cultures is low, serologic assays are poorly standardized [52], and PCR is not generally available [27]. Because only semiquantitative analysis of lower respiratory secretions were available, differentiation between microbiologically definite and probable pneumonia was not possible in all patients. Conversely, because ALI/ARDS also has noninfectious etiologies (aspiration, organizing pneumonia, transfusion-induced lung injury (TRALI), acute eosinophilic pneumonia, diffuse alveolar hemorrhage, and other interstitial lung diseases), we believe that isolation of a microbiologic agent strengthened the diagnosis of infectious pneumonia and prevented the inclusion of noninfectious lung diseases that might have been included by using only the clinical definition.

Second, because we matched by pathogen, we could not properly examine the influence of specific pathogen and immune status on ALI development. It is likely that the results would be different for specific viral (RSV, CMV) or fungal (PCP) pathogens, but limited control data precludes the analysis (because clinicians rarely test immunocompetent patients for these pathogens). Conversely, matching by specific pathogen supports the findings that inappropriate antimicrobial treatment is an independent predictor of ALI and not just a marker of having an atypical pathogen.

Third, differentiation between severe bilateral pneumonia and ALI/ARDS may be challenging. Sensitivity and specificity of clinical assessment compared with a pathologic finding of diffuse alveolar damage (DAD) was found to be poor, with the weakest correlation in pneumonia patients [53].

Fourth, we used the pneumonia severity index as a composite measure of baseline severity of illness in all patients. The PSI has been validated in CAP and HCAP but not HAP patients. However, the results were similar when adjusted for baseline imbalances (shock, SpO_2_/FiO_2_) instead of PSI.

Finally, a limitation of our study is inherent in its retrospective design.

## Conclusions

In conclusion, to our knowledge, this is the first study to assess the relation between ALI and specific respiratory pathogens isolated from patients with pneumonia. The results show that patients with confirmed infectious pneumonia are at a high risk of ALI, especially those with certain types of fungal and viral pneumonias. No difference was found in the occurrence of ALI among the most common bacterial pathogens, suggesting other possible mechanisms that may promote the development of ALI, aside from pathogen characteristics. Potentially modifiable health-care delivery factors such as antibiotic therapy and transfusion pose significant risk and provide important targets for ALI prevention.

## Key messages

• ALI development is common among hospitalized pneumonia patients with positive microbiology.

• The development of ALI among patients hospitalized with infectious pneumonia varies among pulmonary pathogens and is associated with increased mortality.

• Inappropriate initial antimicrobial treatment and transfusion of blood products are modifiable independent predictors of ALI development in pneumonia patients.

## Abbreviations

ALI: Acute lung injury; APACHE: Acute Physiology and Chronic Health Evaluation; BMI: body mass index; CAP: community-acquired pneumonia; CI: confidence interval; ICD-9-CM: International Classification of Diseases: 9th Rev.: Clinical Modification; ICU: intensive care unit; IQR: interquartile range; HCAP: health-care-associated pneumonia; HAP: hospital-acquired pneumonia; OR: odds ratio; PSI: Pneumonia Severity Index score; TRALI: transfusion-induced lung injury.

## Competing interests

The authors declare that they have no competing interests.

## Authors' contributions

Study concept and design, MK and OG; acquisition of data, GL, KML, LT, JV, AA, and MK; analysis and interpretation of data, MK, GL, and OG; drafting of the manuscript, MK and OG; critical revision of the manuscript for important intellectual content, LB and JR; statistical analysis, AH, GL, and MK.

## Supplementary Material

Additional file 1 **Outcomes of ALI cases and matched controls**. Table describing the differences in hospital length of stay, duration of mechanical ventilation, and hospital mortality between ALI cases and matched controls. Click here for file
