# Supplementary material for: Risk factors for the development of acute lung injury in patients with infectious pneumonia
Source: Crit Care. 2012 Mar 14;16(2):R46. doi: 10.1186/cc11247 (PMC3568742; doi:10.1186/cc11247)
Supplement: Additional file 1 — Outcomes of ALI cases and matched controls. Table describing the differences in hospital length of stay, duration of mechanical ventilation, and hospital mortality between ALI cases and matched controls. [file cc11247-S1.DOC]

**Outcomes of ALI cases and matched controls**

|  | **ALI cases**  **N=112** | **Controls**  **N=112** | **P value** |
| --- | --- | --- | --- |
| Hospital length of stay  Median (IQR) | 20.6 (12.1, 33.4) | 8.5 (5.0, 14.7) | <.001* |
| Duration of mechanical ventilation  Median (IQR) | 8.5 (6.7-20.2) | 2.5 (0.6-4.7) | 0.009 |
| Hospital mortality  N (%) | 47 (42.0) | 11 (9.8) | <.001* |

Abbreviations:ALI- Acute lung injury, IQR- Interquartile range,
